# Surgical Process Modeling for Open Spinal Surgeries

**DOI:** 10.3389/fsurg.2021.776945

**Published:** 2022-01-25

**Authors:** Fabio Carrillo, Hooman Esfandiari, Sandro Müller, Marco von Atzigen, Aidana Massalimova, Daniel Suter, Christoph J. Laux, José M. Spirig, Mazda Farshad, Philipp Fürnstahl

**Affiliations:** ^1^Research in Orthopedic Computer Science, Balgrist University Hospital, University of Zurich, Zurich, Switzerland; ^2^Laboratory for Orthopaedic Biomechanics, Institute for Biomechanics, Swiss Federal Institute of Technology (ETH), Zurich, Switzerland; ^3^Department of Orthopaedics, Balgrist University Hospital, University of Zurich, Zurich, Switzerland

**Keywords:** surgical process modeling, open spinal surgery, spinal fusion, spinal instrumentation, pedicle screw, top-down modeling, bottom-up modeling

## Abstract

Modern operating rooms are becoming increasingly advanced thanks to the emerging medical technologies and cutting-edge surgical techniques. Current surgeries are transitioning into complex processes that involve information and actions from multiple resources. When designing context-aware medical technologies for a given intervention, it is of utmost importance to have a deep understanding of the underlying surgical process. This is essential to develop technologies that can correctly address the clinical needs and can adapt to the existing workflow. Surgical Process Modeling (SPM) is a relatively recent discipline that focuses on achieving a profound understanding of the surgical workflow and providing a model that explains the elements of a given surgery as well as their sequence and hierarchy, both in quantitative and qualitative manner. To date, a significant body of work has been dedicated to the development of comprehensive SPMs for minimally invasive baroscopic and endoscopic surgeries, while such models are missing for open spinal surgeries. In this paper, we provide SPMs common open spinal interventions in orthopedics. Direct video observations of surgeries conducted in our institution were used to derive temporal and transitional information about the surgical activities. This information was later used to develop detailed SPMs that modeled different primary surgical steps and highlighted the frequency of transitions between the surgical activities made within each step. Given the recent emersion of advanced techniques that are tailored to open spinal surgeries (e.g., artificial intelligence methods for intraoperative guidance and navigation), we believe that the SPMs provided in this study can serve as the basis for further advancement of next-generation algorithms dedicated to open spinal interventions that require a profound understanding of the surgical workflow (e.g., automatic surgical activity recognition and surgical skill evaluation). Furthermore, the models provided in this study can potentially benefit the clinical community through standardization of the surgery, which is essential for surgical training.

## Introduction

With the advent of new medical technologies, surgical interventions are increasingly becoming sophisticated and specialization of physicians is more abundant (1). As projected in (2), with the introduction of Computer Assisted Surgery (CAS) (3), robotic surgery (4), medical augmented reality (5), and medical Artificial Intelligence (AI) (6), the average Operating Room (OR) today involves different types of digital equipment, information, staff, and processes that collectively aim at improving the patient outcome. As a newly emerging field, “Surgical Data Science,” has been recently defined that aims at “improving the quality of interventional healthcare and its value through capturing, organization, analysis, and modeling of data” (7).

Although surgeries are complex processes, which involve numerous steps, tasks, and actions, surgeries of the same kind are commonly performed with a rather similar and reproducible workflow (8). Many of the modern surgical technologies rely on a proper understanding of the aforementioned surgical workflow and appropriate “models” that highlight the relationships between different interactions within the workflow. Deep understanding of the surgical workflow is crucial when designing context-aware medical technologies that can adapt to the underlying surgical action at any given time. Context-awareness of medical technologies has been noted as a criteria that can increase the operational efficiency (9). Furthermore, standardized models that accurately depict the surgical workflow are essential for surgical training and education (10, 11), OR management (12), and treatment quality evaluation (13).

There is a notable body of literature dedicated to the derivation of models that can capture the surgical workflow based on different sensory observations (i.e., Surgical Process Modeling, SPM). As stated in (14), a surgical process model can be defined as “a set of sequential and parallel activities, executed by clinical and technical team members with different expertise, through preparing and using equipment and tools with the ultimate goal of high-quality treatment of a patient without complications.” In general, the SPM of a given operation can be acquired through a bottom-up or a top-down approach (15). In a top-down modeling strategy, the entire operation is observed and later broken down into different steps. Contrary, the bottom-up approach starts by first defining the individual steps and then finding the inter-connections between them, and linking them until the entire operation is represented. Extensive prior work exists on surgical process modeling and surgical activity recognition that are tailored to minimally invasive laparoscopic surgeries given the readily available endoscopic camera feed that can be used for activity recognition purposes. Dynamic time warping methods were used in (16, 17) to detect the usage of laparoscopic tools visible in the endoscopic camera videos. Feature-based surgical action recognition methods have also been introduced that rely on hand-crafted features extracted from the video feed [e.g., (18)]. More recently, deep learning methods have been proposed that can identify the surgical tasks and phases based on Convolutional Neural Network (CNN) principles in an end-to-end fashion without the need for derivation of hand-crafted features, which can provide superior accuracies (19–21).

Despite the recent technological advances that are tailored to orthopedic surgeries [e.g., (22–25)], to the best of our knowledge, there is a general scarcity of publications that focus on delivering SPMs for such interventions, specifically for ubiquitous open operations. Hence the primary objective of the herein study is to develop a method for creation of a standard SPM for such surgeries.

Compared to other orthopedic pathologies, low back pain can be noted as one of the most prevalent conditions that is present in two thirds of adults once in their life (26) and is among the leading causes of years lived with disability (27–29) associated with prominent socio-economic impacts (30) such as decreased quality of life (31) and early retirement (32). While conservative treatment options exist for patients with mild symptoms (33, 34), instrumented spinal fusion remains the baseline method of treatment for more severe cases of degenerative disk disease with failed conservative treatment (35). Additionally, spinal fusion is the preferred surgical treatment for selected spinal fractures (36), spinal infections (37), and spinal deformity corrections (38) and congenital spinal anomalies (39).

Spinal instrumentation (i.e., spondylodesis) is a common intervention during which pedicle screw implants are placed within the pedicle corridor of each vertebra to provide anchoring support for the interconnecting longitudinal rods (40). Due to the proximity of the anatomy to spinal cord, nerves and blood vessels, this is a technically demanding procedure involving a complex range of surgical actions (41). Screw breaches can happen and are associated with potential complications such as dural lesions, irritation of the nerve root, and other neurological damages in as high as 2, 4, and 3% of the inserted pedicle screws in the thoracolumbar region respectively (42). Such misplacements can result in up to a 6% rate of revision surgeries as previously reported (43).

Although different open spinal interventions achieve the desired patient outcome through different means (e.g., decompression of neural structures, intervertebral cage insertion for spinal fusion, osteotomy for deformity correction, fracture reduction, and tumor debulking or resection) spinal instrumentation can be noted as the underlying process that is utilized mostly in the aforementioned surgeries.

With the adoption of newer surgical techniques, the perioperative complication rate associated with spinal instrumentation has decreased; however, challenges such as dural tears and neurological deficits can still occur (44) due to the complexity of the procedure. Despite the recent advent of modern computer assisted navigation systems for the aforementioned interventions, it was reported in a world-wide survey that only 11% of the surgeries make use of such systems and the majority of the procedures are performed in an “free-hand” fashion (45) (in contrast to operations performed through the use of computer assisted surgery). Non-navigated open procedures are proven to be associated with a low rate of pedicle screw insertion accuracy, with a median accuracy of 86.6% (46).

Considering the abovementioned lack of comprehensive SPMs for orthopedic surgery and the ubiquity of open spinal surgeries, the focus of this work is to develop a SPM that can capture the common surgical elements performed in such interventions and the inter-connections between the events. To that end, we provide first the theoretical basis and ontological background on which our work is built on. Afterwards, we describe the data collection process, and we elaborate on the model creation steps followed for the generation of the SPMs. The SPMs will be developed in a way that they are capable of encompassing not only different granularity levels and event frequencies, but also the possibility of including unexpected deviations in the surgical workflow. The end goal of the resulting SPMs is to serve as the basis for development of future automatic assistive technologies specific to open spine surgeries (e.g., surgical activity recognition and phase detection based on the emerging AI algorithms). Furthermore, the proposed SPMs can be used for surgical training, activity monitoring, and procedure standardization.

## Methods

The following sections will provide a detailed overview into the development of the SPMs for open spinal fusion surgery. First, in Ontology and Deductive Modeling of SPM for Open Spinal Surgeries, we provide the theoretical basis and ontological background of the SPMs developed in our work, including the nomenclature and the terminology used throughout the paper. Afterwards, in Data Collection and Annotation we explain the data collection and analysis process of the surgical workflow for the open spinal surgeries, and finally, in Creation and Refinement of the SPM for Spinal Surgery we describe the model creation and refinement process of the SPM based on the collected data.

### Ontology and Deductive Modeling of SPM for Open Spinal Surgeries

There exist several works that focus on the definition and the implementation of SPMs (14). While efforts have been made for standardization of SPM ontology definition (47), there exist a wide range of techniques that are tailored to a specific type of intervention [e.g., (48–50)]. Furthermore, as stated in (51), there are multiple definitions of modeling granularity levels available in the prior-art with no consensus across different domains. To this end, we have chosen a combination of recognized modeling schemes presented in (15, 52, 53), which are among the most widely used approaches to date that describe a SPM based on 6 granularity levels (μ) that correspond to different hierarchical elements, with μ ϵ [0,5]. The highest granularity level (μ = 5) encompasses the surgical procedure itself, therefore SPM will often have only one element at this granularity level. In the model described by (52), surgical procedures are further divided into steps (μ = 4), sub-steps (μ = 3), tasks (μ = 2), sub-tasks (μ = 1) and down to the lowest hierarchical level (μ = 0), which describes each motion performed by the surgeon. This model provides information about the granularity level and hierarchy but does not provide other important parameters, such as temporal information, technologies used, structures involved, and participants. The SPM technique developed in (53) presented a model, which includes such missing information and is focused on integrating the control systems in the operation room. However, the granularity level used in (53) does not allow for an easy clinical description of the surgical workflow, as the processes are described in a rather technical manner and are not intuitive for clinical setup. Thus, in the herein study we used a modified version of the model presented in (52), with enhancements inspired from (15) and (53).

#### Hierarchical Decomposition and Nomenclature of Surgical Process Models

**Figure 1:**
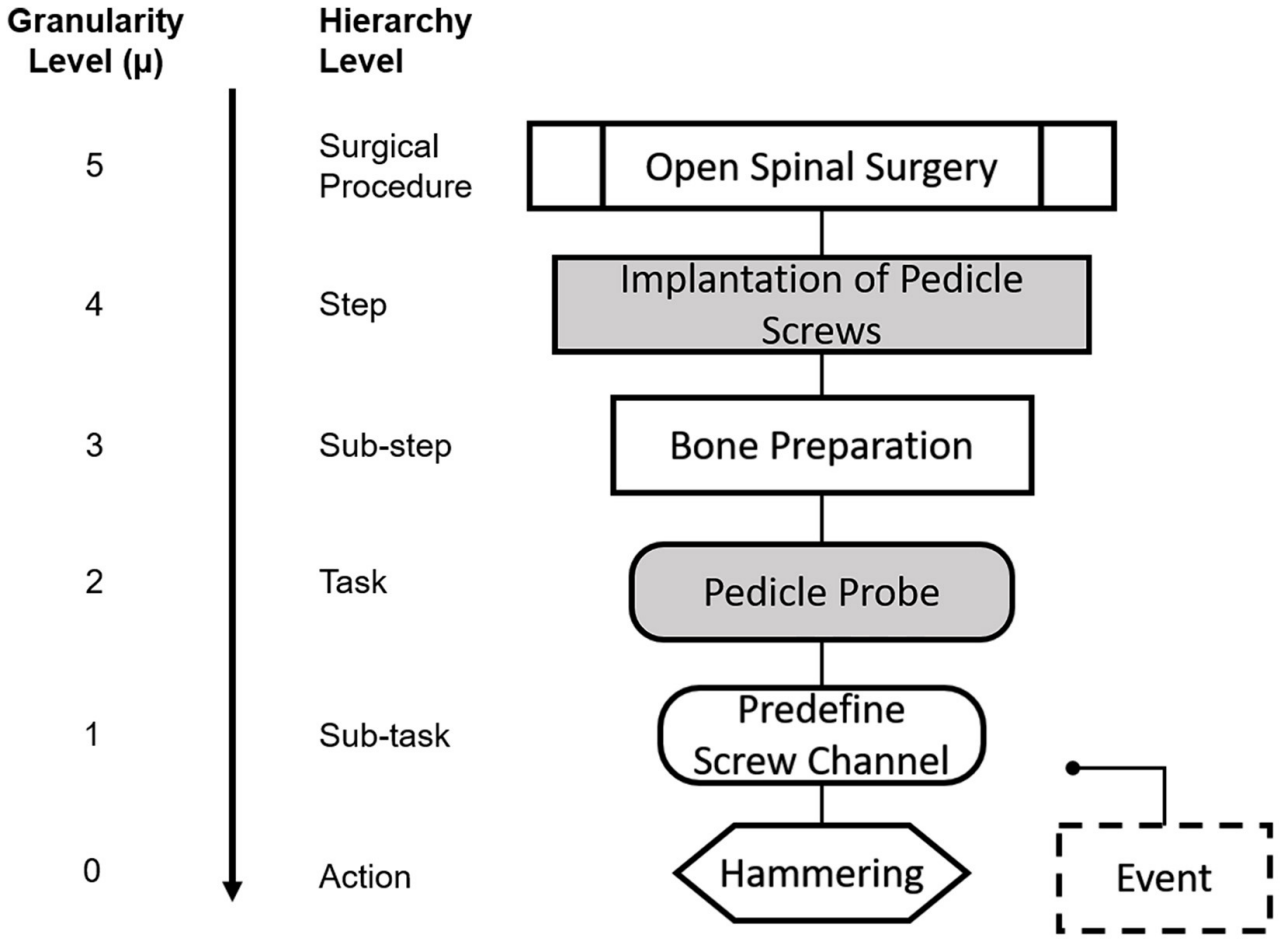
Nomenclature for the Granularity and Hierarchy Levels used in our SPMs. Each elements is depicted with a distintive geometrical shape throughout the manuscript. We show here an example case for each of the given granularity levels. The higher granularity level (μ = 5) correspond to the surgical procedure itself and is depicted as a marked rectangle. μ = 4 and μ = 3 correspond to steps and substeps and are denoted by a filled and a non-filled rectangle, respectively. μ = 2 and μ = 1 correspond to tasks and sub-tasks and are denoted by a filled and non-filled rounded rectangle, respectively. Actions (μ = 0) are denoted by hexagon and events are represented by a dashed rectangle.

We propose a SPM with specific shapes and shadings for each hierarchical level as shown in Figure 1, so that we can easily identify different levels of granularity. This is required in order to capture the highly dynamic changes of surgeries within the SPM. During the normal surgical workflow, the surgeon often revisits one of the previous tasks or subtasks while still focusing on the same surgical step, meaning that the surgeon could complete elements of different granularity levels. For example, the surgeon might decide to cement the bone (μ = 0) while inserting a pedicle screw (μ = 3), because the quality of the bone is not as expected and the screw purchase might be otherwise compromised. In order to capture these changes throughout hierarchical levels, we needed to allow for our SPMs to operate at different granularity levels.

As indicated in Figure 1, granularity level μ = 5 corresponds to the surgical procedure itself:, open spinal surgery (e.g., spinal fusion surgery), as presented also in (52). This surgical procedure is subsequently decomposed into surgical steps (μ = 4). In our model, the surgical steps correspond to the set of standardized steps in the clinical literature of each procedure, with the purpose of maximizing compatibility with the clinical workflow, and to facilitate the identification of the different steps during the surgical process analysis. Thus, granularity level μ = 4 is associated to each of the steps that define the spinal fusion procedure from a high-level perspective.

In order to include a more dynamic representation of the real workflow of the surgery, steps must be further divided into lower levels of granularity. Hence, in our model, surgical steps are further decomposed into sub-steps (μ = 3). These sub-steps are defined as the collection of smaller units that can be associated to surgical checkpoints during the intervention. To this end, technical manuals provided by spinal fusion implant manufacturers (USS Universal Spine System, OPAL, MATRIX Spine System, Synthes GmbH, Oberdorf, Switzerland) were a valuable source that allowed a better insight into the definition of the sub-steps and tools used in the surgical procedure. For instance, in the example case presented in Figure 1, for the step “Implantation of pedicle screws”, there are two surgical sub-units or sub-steps that must be executed for the overarching step to be completed. In a first sub-step, the bone must be prepared for the screw insertion, and in a second sub-step the insertion of the screw itself is performed. This level of granularity also has the attribute of repetition, meaning that a sub-step could be done several times before moving on to the next one.

Subsequently, sub-steps can be further split into surgical tasks and sub-tasks with μ = 2 and μ = 1, respectively. Surgical tasks and sub-tasks are smaller workflow elements that are needed for the completion of a sub-step and that can be associated to specific techniques or tools during the surgery. The difference between a task and a sub-task, is that tasks are directly related to a specific sub-step in the hierarchy, whereas the same sub-task can be found across different sub-steps. Using again the example of Figure 1, for the sub-step “Bone preparation,” the task “Pedicle probe” is needed, which entails predefining the screw channel and performing hemostasis to stop bleeding (sub-tasks, μ = 2). The latter is not exclusive to the bone preparation step, and it is in fact performed across different steps and sub-steps throughout the surgical procedure. The lowest hierarchical level “Action” with granularity μ = 0 included in our model defines the surgical actions, which refer to specific workflow units related to the action performed by the surgeon, i.e., hammering, cementing, cutting, etc.

We have also included some enhancements to our SPM analysis inspired by the model in (15): First, the surgical tool used is indicated within the name of the “Action” when appropriate. Second, we have included an additional element with granularity levels μ ≤ 1, denominated “Event” and it is used to represent unexpected deviations in the standard surgical protocol that might happen more than once.

#### Deductive SPM for Open Spinal Surgeries

**Figure 2:**
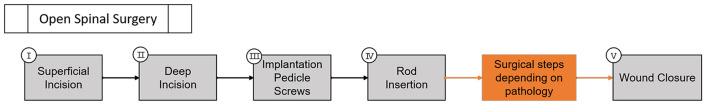
Deductive SPM for open spinal surgery at the step level (μ = 4), showing the 5 main surgical steps identified for open spinal surgery: (I) superficial incision, (II) deep incision, (III) implantation of pedicle screws, (IV) rod insertion, and (V) wound closure. Additional pathology-specific steps are depicted in orange and are usually located between steps (IV) and (V).

The systematic analysis of a surgical process required an in-depth insight into the surgical procedure and a sufficient understanding of the main steps, the used instruments, and technical aspects of the surgeries. Therefore, before the analysis and creation of the SPMs from empirical data, a deductive SPM of the surgical procedure was created at the step level (μ = 4) and is shown in Figure 2. Five surgical steps were identified from the available clinical literature: (I) superficial incision, (II) deep incision, (III) implantation of pedicle screws, (IV) rod insertion, and (V) wound closure. Considering the variations amongst different surgical techniques that are dedicated to specific conditions (e.g., trauma, deformity correction, tumor surgery, etc.) an intermediate step between steps (IV) and (V) was identified and highlighted in a different color in Figure 2. Given that almost all open spinal interventions share the steps (I)–(V) in common, this work only focuses on providing detailed SPMs for those steps. These steps were proposed based on orthopedic textbooks (54) and online resources with process descriptions and surgical guidelines of spinal fusion procedures (www.orthobullets.com). Furthermore, consultations with surgery specialists were made to ensure the adequacy of the initial deductive model (See Appendix). This deductive SPM at the step level was validated using two video recordings of spinal fusion surgery, recorded at our institution as part of the standard clinical procedure (see Data Collection and Annotation). In particular, the workflow at the hierarchical level of surgical steps is conceived as successive, meaning that the steps are expected to follow a forward direction from the first step, up to the last one.

As stated before, in our SPM, the connection between elements can happen across different hierarchical levels and this evolution between different elements of the SPM is denominated as “Transition” and is represented with a solid arrow. These transitions indicate the standard or expected workflow. Transitions that occur due to repetition, or that happen as consequence of deviations of the standard workflow are depicted with a dashed arrow.

Through iterative top-down analyses of surgical procedures and bottom-up analyses of tool motions, we provided detailed decomposition of the procedures down to the level of actions. From observable surgical elements based on the data collection (Data Collection and Annotation), we have operationally defined the beginnings and endings of surgical steps, sub-steps, tasks, sub-tasks and actions. We have recorded the process from the main surgeon's perspective, therefore the target participant was always the same. In terms of the anatomy, as the SPM is specific for the spine, the body part was always the vertebrae.

### Data Collection and Annotation

We collected videos from open spinal operations performed at our clinic on 24 patients, between April and July 2019. We included videos captured from spinal fusion operations performed mainly in the lower thoracic and lumbosacral region (Th11-S1), which are performed more commonly. The included operations were performed to account for the following pathologies (some patients had multiple pathologies): fracture, degenerative disc disease, foraminal stenosis, spinal canal stenosis and spondylolisthesis. Each operation was performed by two surgeons and data from a total of 5 surgeons were included. A total of 157 pedicle screws and 30 interconnecting longitudinal rods were inserted.

Given the practical limitations in recording the entire duration of the surgeries, we identified the step “implantation of pedicle screws” to have the highest priority given its inherent surgical complexity. Therefore, we ensured that all the recordings at least included the entirety of this step and possibly more of the surgical steps.

We collected intraoperative video recordings of the aforementioned spinal operations using 3 different camera systems: a flexible crane camera (custom-made model for our hospital; Medical-Polecam, Apoint Film GmbH, Switzerland), a fixed camera integrated into the operation room's surgical light (truvidia wireless camera, 3.8–38 mm focal length, 1,080p resolution; Trumpf Medical, Germany) and an eye-tracking head-mounted camera (SMI eye tracking glasses; iMotions GmbH, Bern, Switzerland). The intraoperative videos acquired from the crane and fixed camera were previously recorded for training or quality control purposes in the frame of the standard surgical procedure. The videos captured using the eye-tracking camera were acquired within the frame of a related project within our group (BASEC No. 2018-00533). In total, we collected the video data from 24 spinal fusion surgeries performed at our institution resulting in roughly 40 h and 30 min of video footage. Information about screw insertion and fluoroscopy times was obtained from the operation protocol of each corresponding surgery. We also noted auxiliary information about the relevant intraoperative events, which could not be inferred from the video recordings, namely: the position of the two main surgeons (which surgeon was on which side of the patient and who performed which surgical steps), and unexpected events leading to deviations in the surgical procedure.

We investigated all the recordings to find the starting and the ending points in which all the primary surgical elements took place. The starting point of a video was set at the localization of the target spine levels and the dorsomedial skin incision; thereafter, the ending point was set at the wound closure. Later, we temporally annotated all of the events that were observable in the video data up to granularity level μ = 0 (if necessary for the respective surgical step). For each operation, all the transitions between the observed elements were recorded in a spreadsheet, which allowed to keep track of their frequency.

### Creation and Refinement of the SPM for Spinal Surgery

**Figure 3:**
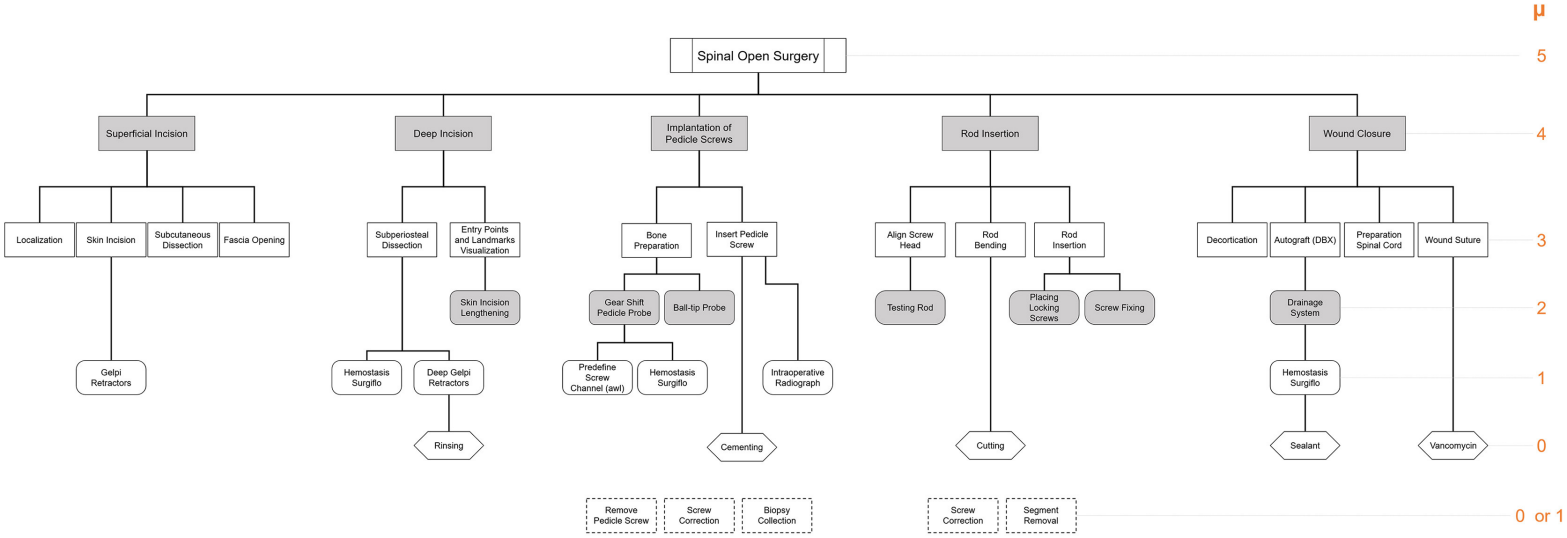
Hierarchical decomposition for the 5 identified surgical steps of the open spinal surgery using the nomenclature defined in Figure 1. Events are represented by a dashed square and are not linked to the rest of the tree, but they are located directly below the corresponding surgical steps where they are expected to happen. Level of granularity is indicated in orange on the right-hand side of the figure.

Having a set of recordings for the surgical procedures is one of the first steps for the analysis and generation of the SPMs. Manual labeling of the videos data and tracking of the transitions between the different states of the surgery allowed for working out the relationship between all the elements of the surgical workflow. For the exploration of several recorded processes and general overview of all the elements of the intervention under analysis, the hierarchical decomposition of the procedure is needed. Based on the surgical steps of the deductive SPM presented in Figure 2, and the aforementioned ontology, we generated the hierarchical decomposition for spinal fusion surgeries, and its graphical representation is presented in Figure 3. The upper most level (μ = 5) is dictated by the surgical procedure itself, which is then branched into the identified surgical steps (μ = 4). Subsequently, the hierarchical tree was iteratively refined using the information of the spreadsheet generated based on the annotated videos, the temporal information about the surgical task (0 ≤ μ ≤ 4), and the transitions between the elements.

In a first iteration, each surgical step was extended into their corresponding sub-steps (μ = 3). In case the sub-step level was not sufficient to describe the corresponding workflow of the surgery, it was further decomposed into tasks (μ = 2). The iterative process was performed until the granularity level was sufficient to describe the surgical element in question (from a clinical perspective), or until the lowest granularity level was reached (μ = 0). If an unexpected event happened during one of the surgical recordings, which is not normally part of the operation or which had to be performed because of a deviation of a surgical element, the event was noted and the number of transitions to that event was measured; however, the element was not linked to the rest of the hierarchical tree. These events are represented by a dashed square and are located directly below the corresponding surgical steps where they have been identified. The hierarchical decomposition of Figure 3 is used as a guide to generate the individual SPM of each of the surgical steps of the spinal fusion procedure shown in Figure 2.

The final SPMs presented in this study have a granularity level μ ≤ 3, which includes sub-steps, tasks, sub-tasks, and actions. For the generation of each SPM, an iterative approach was followed. In a first phase and for each surgical step, all surgical elements with μ = 3 were identified from the hierarchical tree (Figure 3). Subsequently, the transition between the sub-steps was set using the annotated information in the spreadsheet. After each iteration, the number of transitions between the same sub-steps among all recorded operations was updated. The total number of transitions between every two elements is indicated with an integer above the connecting arrows. As stated previously, transitions that occur due to repetition, or that happen as consequence of deviations of the standard workflow were depicted with a dashed arrow. Iteratively, the transitions between all the workflow elements were updated for each granularity level, down to the level of actions. The final SPMs are given in the form of digital diagrams and were generated using the diagramming software Microsoft Visio (Creative Cloud, Microsoft Corporation, Redmond, Washington, U.S., 2020) and the tools for workflow drawing provided by Microsoft Power Point (Microsoft Office 2020, Microsoft Corporation, Redmond, Washington, U.S.). Finally, all of the generated SPMs were presented to one of the chief spine surgeons at our institution, who evaluated the quality and accuracy of the models and confirmed their validity.

## Results

Based on our collected data, the operations took an average of 2.5 h and were performed on an average of 3–4 spinal segments. For each of the 5 steps, we have generated individual SPMs with a minimum granularity level of μ = 0 wherever it was needed. Each step was further divided according to the process described in the methodology.

As stated earlier, the *implantation of pedicle screws* step was included in all of the 24 recordings of the surgeries; however, a lower number of case recordings were present for the rest of the steps. An overview of the total number of cases for which a given surgical step was recorded is presented in Table 1.

**Table 1:** Overview of the total number of cases recorded for a given surgical step.

**Surgical step**	**Number of cases recorded**
Superficial incision	11
Deep incision	22
Screw Implantation	24
Rod-Insertion	15
Wound Closure	12

**Figure 4:**
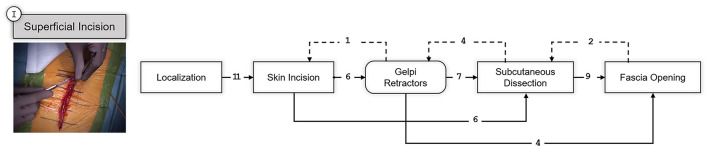
SPM for the Superficial Incision step. A snapshot of the specific part of the surgery involving this step is shown on the left. Transitions are indicated with an arrow and the number of transitions between the elements are indicated above the arrow with an integer number. Dashed arrows indicate repetitions or deviations from the standard workflow.

The first step was the superficial incision of the tissue over the vertebral segments to be fused. The resulting SPM for this step is shown in Figure 4.

**Figure 5:**
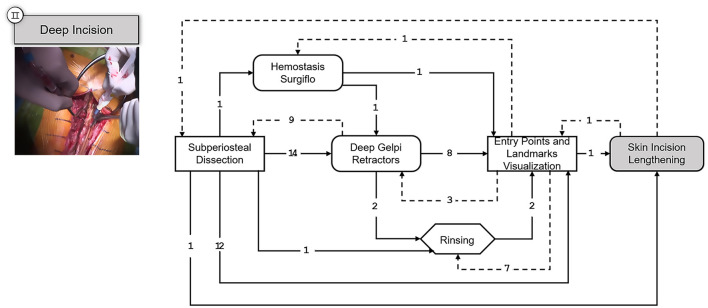
SPM for the Deep Incision step. A snapshot of the specific part of the surgery involving this step is shown on the upper left corner. Transitions are indicated with an arrow and the number of transitions between the elements are indicated above the arrow with an integer number. Dashed arrows indicate repetitions or deviations from the standard workflow.

The following step, corresponds to the deep incision of the musculature after opening the muscle fascia so that the vertebral bodies are visualized. The obtained SPM is depicted in Figure 5.

**Figure 6:**
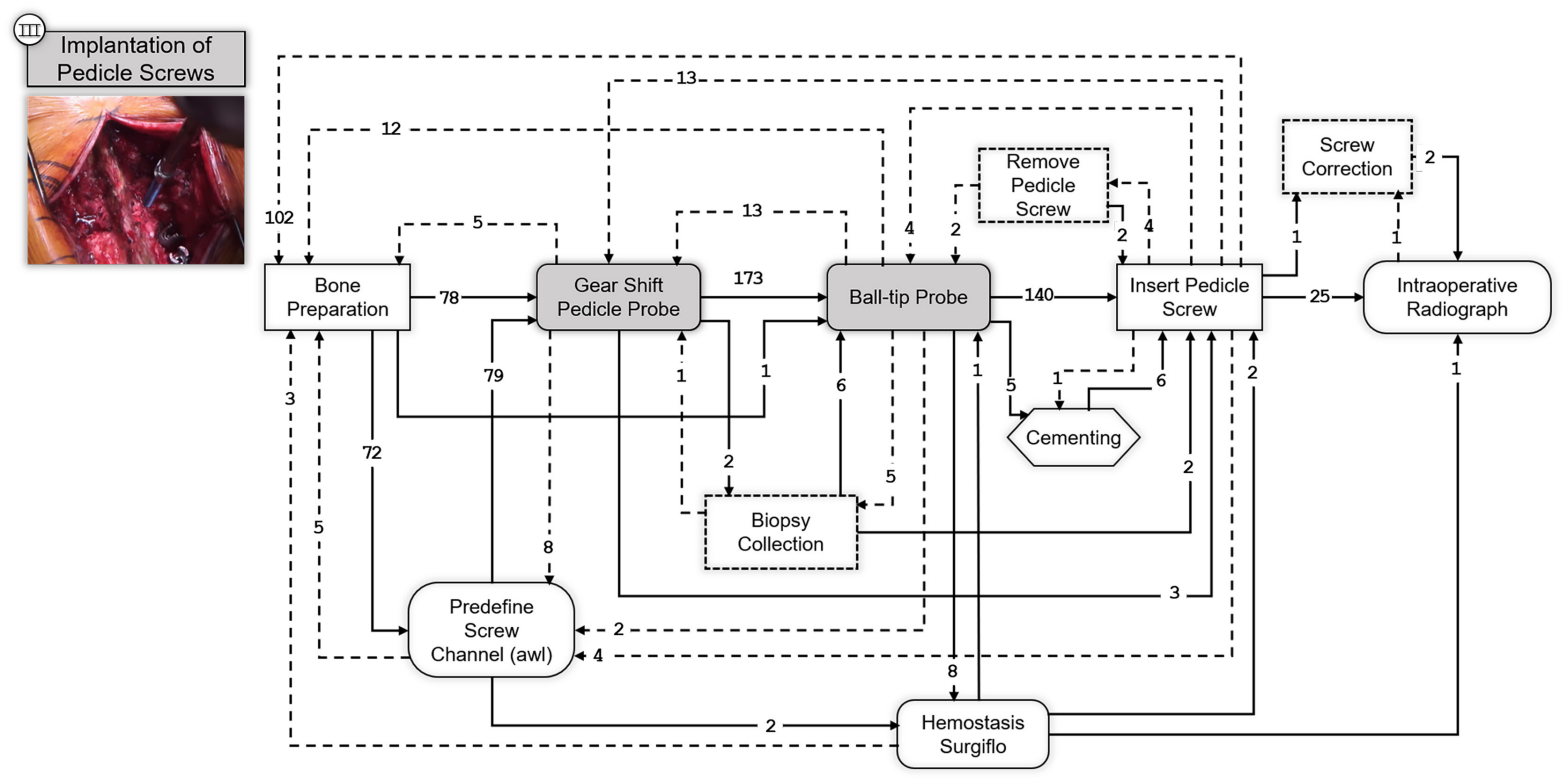
SPM for the Implantation of Pedicle Screws step. A snapshot of the specific part of the surgery involving this step is shown on the upper left corner. Transitions are indicated with an arrow and the number of transitions between the elements are indicated above the arrow with an integer number. Dashed arrows indicate repetitions or deviations from the standard workflow.

**Figure 7:**
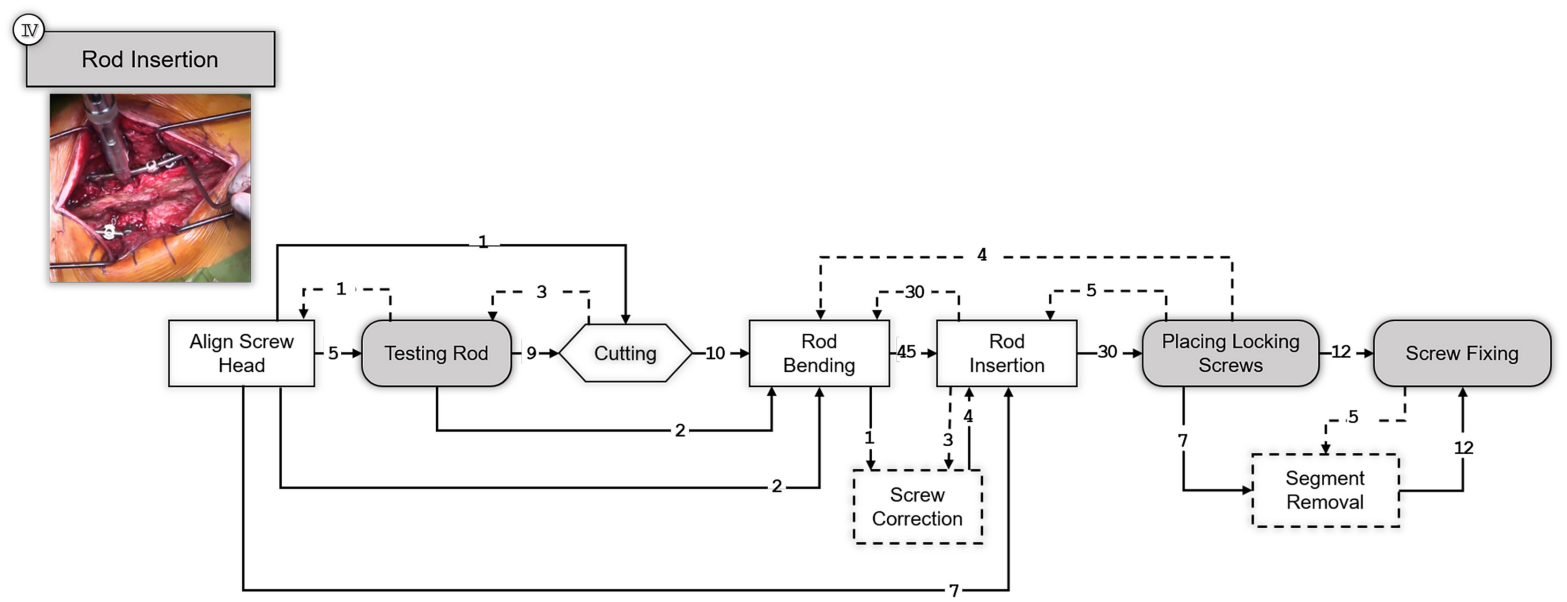
SPM for the Rod Insertion step. A snapshot illustrating the specific surgical step is shown on the upper left corner. Transitions are indicated with an arrow and the number of transitions between the elements are indicated above the arrow with an integer number. Dashed arrows indicate repetitions or deviations from the standard workflow.

In the third and fourth step, the intended instrumentation of the pedicle screw system and the insertion of the connecting rods takes place, Figures 6, 7 respectively. As stated before, these two steps are optionally followed by various procedures depending on the underlying conditions. Depending on the operation, procedures for decompression of the spinal canal or spinal nerves via various techniques can be performed and cages can be inserted for anterior stabilization of the spinal column (TLIF and PLIF). Also, pedicle subtraction osteotomies (PSO) might be performed at this point. However, such condition-specific steps are excluded in this work to provide SPMs that can generalize to a wider range of open spinal surgeries.

**Figure 8:**
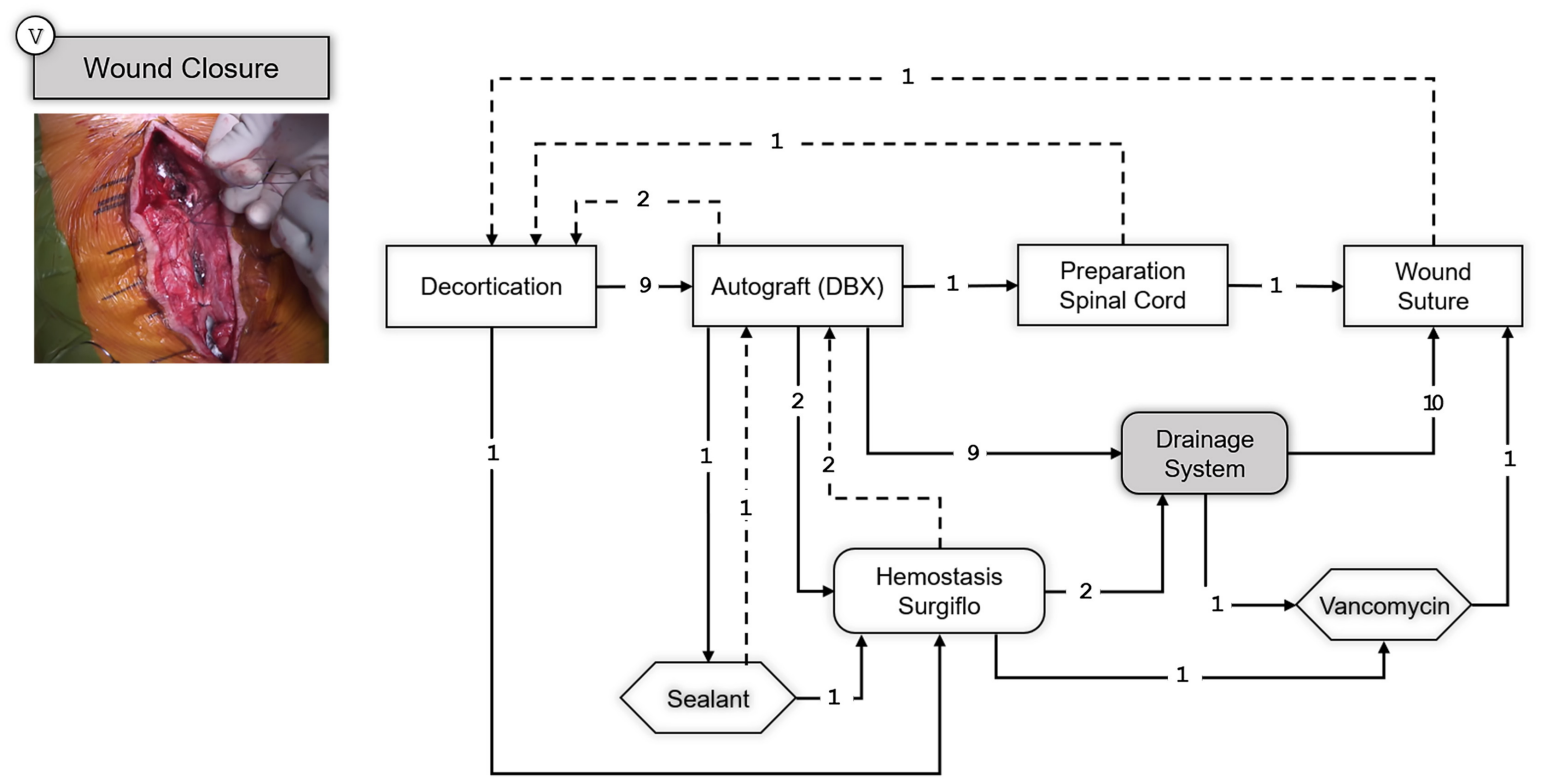
SPM for the Wound Closure step. A snapshot of the specific part of the surgery illustrating this step is shown on the upper left corner. Transitions are indicated with an arrow and the number of transitions between the elements are indicated above the arrow with an integer number. Dashed arrows indicate repetitions or deviations from the standard workflow.

Finally, the SPM of the last step of the surgical procedure is shown in Figure 8, corresponding to the wound closure.

## Discussion

With the recent advent of advanced medical technologies and the ever-increasing surgical workflow complexity, it is important to derive comprehensive surgical process models that can capture the activities conducted in a common surgical intervention and the inter-relationships between the events. Such models are of utmost importance for objectives such as: standardization of the interventions, surgical training and communication, surgical team workload analysis and operating room management, and development of algorithms that are focused on applications such as: surgical activity recognition [e.g. (55)], surgical skill evaluation [e.g. (56)], prediction of next surgical tasks [e.g. (57)], intervention time prediction [e.g. (58)]. Despite the abundance of methods and algorithms for generating SPMs for laparoscopic and endoscopic procedures [e.g. (49)], modeling of open spinal interventions remains an underrepresented task. Therefore, in this study, we provided an SPM dedicated to spinal fusion surgery through annotation and analysis of direct video observations captured during such operations.

An analogy similar to (52) was used to classify the hierarchy of the surgical elements. In the pre-modeling phase, we relied on the available intervention-specific literature [e.g., (54)] to derive a high-level model of the surgeries up to granularity level μ = 4. The process models were then further expanded up to the action level (μ = 0) based on manual annotation and analysis of the captured video observations. We encountered challenges when analyzing recordings captured by the fixed OR light camera. First, the view was often obscured by the surgeons' heads or due to the moving of the operating light. This resulted in difficulty in assessing the position of the anatomy only based on the static two-dimensional video feed provided by this camera assembly. In addition, most operations were performed within a large operating area, and the operating table was often moved up and down, making it harder to observe the surgical elements based on the fixed OR light camera feed. We overcame these challenges by using the recordings that were available from the two other camera modalities. The crane camera footage provided more viewing angles since it was manually placed at a position to provide a view with a low level of obscurity, usually at an angle between the OR lights, or at an oblique angle from cranial or caudal directions, as well as from oblique lateral direction. Using the control unit on the embedded 3-axis remote camera head of the crane camera, the position was altered in the recordings to achieve a clear view on to the anatomy. Additionally, the zoom function on this camera platform provided both wide-range overview shots and clos-up views. The video recordings from the crane camera provided a more reliable view of the operating area, the anatomy at which the operation was performed, the forearms of the surgeon, and the instruments used. However, those videos provided limited information about events that occurred outside the surgical area (e.g., cutting and bending of connecting rods) and interactions between the surgeons and the assistants. Using the video recordings from the eye-tracking head-mounted camera we were able to address these limitations. The first-person view provided additional information about the surgeon's line of sight, which captured many auxiliary surgical elements that were happening outside the operating area (e.g., fluoroscopy, interaction with the team, viewing the MRI/CT images).

We provided detailed SPMs for each major surgical step that are common in most open spinal surgeries and highlighted the transitions between different surgical elements and the frequencies associated with those transitions. During the analysis of the videos, we noticed some deviations from the expected surgical workflow, and we included those as events into the generated SPMs. Even though these unexpected events are rather common during surgery, they cannot be classified as a part of a surgical step and are currently difficult to predict. For instance, during spine surgeries, it might be necessary to correct the position of a pedicle screw or to change its length intraoperatively, due to low bone density in the pedicle or to a slight deviation from the afore planned surgery. These events are unavoidable but could be reduced to a minimum with an improved surgical guidance. Therefore, creating SPMs which accounts also for these deviations could eventually allow surgeons to anticipate such errors during the specific steps where they are more prompt to occur.

Knowing beforehand the possible transitions between the surgical steps, sub-steps, task, sub-task, and motions could not only improve the surgical workflow, but it could also assist novice surgeons and less experienced surgical scrub technicians to be better prepared during the surgery.

Following, we would like to discuss some of the limitations of this work. Firstly, it should be noted that although the inter-institution variability was taken into account in the developed SPMs (given that we have analyzed the data from five different spine surgeons), further data is required to improve the generalizability of the developed model to other institutions and surgeons. This is because the herein presented SPMs are closely linked to the clinical setting and to the surgical technique utilized at our clinical institution. If, for instance, a different pedicle screw supplier would be used, this might modify or introduce additional elements into the surgical workflow, which our SPMs do not currently account for. In other words, the inter-clinical variability of the generated SPMs highly depends on the adopted surgical techniques of each operating room. Another limitation of our work was the manual labeling of the video recordings, which is very time consuming and requires surgical and orthopedic knowledge. The quality of the manual video annotations is also subjective to the quality of the video and to the different perspectives available. A possible solution could be the use of a specialized software such as the SWAN-Suite (59), which has proven to be highly accurate when registering and analyzing surgical workflows in several institutions. There exist other video-based algorithms (60–62) that could help in improving the labeling accuracy of the SPM creation process. However, most of the recent data-driven surgical activity recognition methods rely on a proper understanding of a model that describes the underlying surgical 'process'; therefore, the presented SPMs in this study can serve as the basis for the development of such algorithms that can be used in open spinal surgery applications. Finally, the generated SPM in this study was only validated based on the feedback from one surgeon.

Similar validation methods that are based on structured interviews with surgeons have been previously introduced [e.g., (63)]. A more extensive evaluation and validation protocol will contribute to refinement and standardization of the developed SPMs through follow-up studies based on input from multiple surgeons and multiple centers, as well as a technical validation through additional surgery observations with a dedicated SPM Ontology software [e.g., (55, 59, 60)].

## Conclusion

The herein study presents the methodological process through which we generated a series of SPMs corresponding to the most common surgical steps required for open spinal surgeries. For this, we followed a top-down modeling approach for the first theoretical model of the SPM, and a bottom-up granularization approach derived from the video analysis. Combining these two development directions ensured a thorough description of the entire surgical procedure. The resulting SPMs represent a first step into digitizing surgery information and can help to improve the current intraoperative guidance methods, as well as easing the future integration of augmented reality and machine learning approaches into the daily clinical practice.

## Data Availability Statement

The datasets presented in this article are not readily available because according to our institutional guidelines, no patient data can be shared with third parties outside the umbrella of our organization. Requests to access the datasets should be directed to https://rocs.balgrist.ch/en/open-access/.

## Ethics Statement

Ethical review and approval was not required for the study on human participants in accordance with the local legislation and institutional requirements. Written informed consent for participation was not required for this study in accordance with the national legislation and the institutional requirements.

## Author Contributions

PF, MF, JS, CL, HE, FC, MA: conceptualization. MF, JS, PF: resources. HE, FC, SM, AM, DS: methodology. SM, MA: data collection. SM: data annotation. FC, HE: data curation. SM, HE, FC: process model derivation. HE, FC: writing (original draft preparation). PF, HE, FC, MF, JS, CL, SM, MA: writing (review and editing). PF, HE, FC, MA: supervision. DS, CL, HE, FC: project administration. All authors contributed to manuscript revision, read, and approved the submitted version.

## Funding

The work was supported by the Functionally Accurate Robotic Surgery (FAROS) project. This project has received funding from the European Union's Horizon 2020 research and innovation program under grant agreement No 101016985.

## Conflict of Interest

The authors declare that the research was conducted in the absence of any commercial or financial relationships that could be construed as a potential conflict of interest.

## Publisher's Note

All claims expressed in this article are solely those of the authors and do not necessarily represent those of their affiliated organizations, or those of the publisher, the editors and the reviewers. Any product that may be evaluated in this article, or claim that may be made by its manufacturer, is not guaranteed or endorsed by the publisher.

## Appendix

**Table:**

**Surgical Step**	**Description**
**Superficial incision**	**Preparation until the thoracolumbar fascia is reached**.
Preoperative marking	Check if patient has been preoperatively marked at the planned level.
Identification of landmarks	Mark bony prominences (posterior superior iliac spine, iliac crest, spinous processes, inter spinal window) and draw incision line. Ensure with fluoroscopy that correct levels are identified.
Skin incision over the target segments	Incision over the entire planned instrumentation length and through the full thickness of the skin.
Dissection of subcutaneous tissue	Preparation of subcutaneous fat until the thoracolumbar fascia is reached. Insertion of retractors is performed.
Opening of the thoracolumbar fascia	Opening of the thoracolumbar fascia and exposure of spinous process.
**Deep incision**	**Exposure of bony landmarks**
Detach paraspinal muscles	Subperiosteal detachment of the paraspinal musculature with electrocautery/Cobb Elevator. Remain strictly subperiosteal.
Insertion of deep retractors	Insert Deep retractors.
**Implantation screw system (Pedicle screws)**	**Metal implants are placed in the pedicles so that on the one hand they provide optimal stability and on the other hand they do not injure critical soft tissues**.
Bone preparation	Various options to prepare the pedicle for screw insertion (tagged as A,B and C).
Localization of the screw entry point	Localization of the screw entry point using anatomical landmarks. (Pars interarticularis, mamillary process, lateral border of the superior articular facet, mid transverse process).
A.Drilling	Definition of screw trajectory with drill (detailed in the following points).
A.1. Pre-drilling	Direct drilling of the screw channel with a drill.
A.2. Check the screw canal	Use ball tip probe to check for perforations and confirm length of pedicle screw.
B. Insertion of K-wire	Direct drilling of a K-wire as a guide for a cannulated screw.
C. Conventional method	Definition of screw trajectory with awl (detailed in the following points).
C.1. Opening of cortex	Opening the cortex with luer or pointed awl.
C.2. Pre-define screw channel with surgical bone awl (optional)	Use the awl to predefine the screw channel.
C.3. Insert gear shift pedicle probe / pedicle deepening awl	Determine the length and diameter of the required screw using the attached marking and imaging information.
C.4. Check the screw canal	Use ball tip probe to check for perforations and confirm length of pedicle screw.
Insert Pedicle Screw	Insert pedicle screw into predefined channel and maintain the trajectory.
Check screw purchase	Check insertional torque and pull-out resistance.
Check Screw position	Check screw position with intraoperative radiograph.

## References

[1] Stitzenberg KB Sheldon GF. Progressive specialization within general surgery: adding to the complexity of workforce planning. J Am Coll Surg. (2005) 201:925–32. 10.1016/j.jamcollsurg.2005.06.253 16310697

[2] Cleary K Kinsella A Mun SK. OR 2020 workshop report: operating room of the future. Int Congr Ser. (2005) 1281:832–8. 10.1016/j.ics.2005.03.279

[3] Joskowicz L. Computer-aided surgery meets predictive, preventive, and personalized medicine. EPMA J. (2017) 8:1–4. 10.1007/s13167-017-0084-8 28670350 PMC5479310

[4] Herrell SD. Robotic surgery: past, present, and future. In: Su L-M, editor. Atlas of Robotic Urologic Surgery. Cham: Springer International Publishing (2017). p. 459–72.

[5] Negrillo-Cárdenas J Jiménez-Pérez JR Feito FR. The role of virtual and augmented reality in orthopedic trauma surgery: from diagnosis to rehabilitation. Comput Methods Programs Biomed. (2020) 191:105407. 10.1016/j.cmpb.2020.105407 32120088

[6] Hashimoto DA Ward TM Meireles OR. The role of artificial intelligence in surgery. Adv Surg. (2020) 54:89–101. 10.1016/j.yasu.2020.05.010 32713441

[7] Maier-Hein L Vedula SS Speidel S Navab N Kikinis R Park A. Surgical data science for next-generation interventions. Nat Biomed Eng. (2017) 1:691–6. 10.1038/s41551-017-0132-7 31015666

[8] Padoy N Blum T Feussner H Berger M-O Navab N. On-line recognition of surgical activity for monitoring in the operating room. In: Proceedings of the 20th national conference on Innovative applications of artificial intelligence, Vol.3. Chicago, IL: AAAI Press (2008).

[9] Padoy N Blum T Ahmadi SA Feussner H Berger MO Navab N. Statistical modeling and recognition of surgical workflow. Med Image Anal. (2012) 16:632–41. 10.1016/j.media.2010.10.001 21195015

[10] Riffaud L Neumuth T Morandi X Trantakis C Meixensberger J Burgert O. Recording of surgical processes: a study comparing senior and junior neurosurgeons during lumbar disc herniation surgery. Neurosurgery. (2010) 67(2 Suppl Operative):325–32. 10.1227/NEU.0b013e3181f741d7 21099555

[11] Nakawala H Ferrigno G De Momi E. Development of an intelligent surgical training system for thoracentesis. Artif Intell Med. (2018) 84:50–63. 10.1016/j.artmed.2017.10.004 29169646

[12] Liebmann P Neumuth T. Model driven design of workflow schemata for the operating room of the future. In: INFORMATIK 2010 Service Science–Neue Perspektiven für die Informatik. Bonn: GI (2010). p. 419.

[13] Driessen SRC Van Zwet EW Haazebroek P Sandberg EM Blikkendaal MD Twijnstra ARH. A dynamic quality assessment tool for laparoscopic hysterectomy to measure surgical outcomes. Am J Obstet Gynecol. (2016) 215:754.e1–754.e8. 10.1016/j.ajog.2016.07.004 27402052

[14] Gholinejad M Loeve AJ Dankelman J. Surgical process modelling strategies: which method to choose for determining workflow? Minim Invasive Ther Allied Technol. (2019) 28:15. 10.1080/13645706.2019.1591457 30915885

[15] Neumuth T. Surgical process modeling. Innov Surg Sci. (2017) 2:123–37. 10.1515/iss-2017-0005 31579744 PMC6754019

[16] Padoy N Blum T Essa I Feussner H Berger MO Navab N. A boosted segmentation method for surgical workflow analysis. Med Image Comput Comput Assist Interv. (2007) 10(Pt 1):102–9. 10.1007/978-3-540-75757-3_13 18051049

[17] Ahmadi SA Sielhorst T Stauder R Horn M Feussner H Navab N. Recovery of Surgical Workflow Without Explicit Models. In: Larsen R Nielsen M Sporring J, editors. Medical Image Computing and Computer-Assisted Intervention – MICCAI 2006. Berlin, Heidelberg: Springer (2006). p. 420–8. (Lecture Notes in Computer Science) 10.1007/11866565_52 17354918

[18] Zappella L Béjar B Hager G Vidal R. Surgical gesture classification from video and kinematic data. Med Image Anal. (2013) 17:732–45. 10.1016/j.media.2013.04.007 23706754

[19] Twinanda AP Shehata S Mutter D Marescaux J Mathelin M de Padoy N. EndoNet: a deep architecture for recognition tasks on laparoscopic videos. IEEE Trans Med Imaging. (2017) 36:86–97. 10.1109/TMI.2016.2593957 27455522

[20] Bodenstedt S Wagner M Katić D Mietkowski P Mayer B Kenngott H. Unsupervised Temporal Context Learning Using Convolutional Neural Networks for Laparoscopic Workflow Analysis. ArXiv170203684 Cs. (2017). Available online at: http://arxiv.org/abs/1702.03684 (accessed January 12, 2021).

[21] Si C Chen W Wang W Wang L Tan T. An Attention Enhanced Graph Convolutional LSTM Network for Skeleton-Based Action Recognition. ArXiv190209130 Cs. (2019). Available online at: http://arxiv.org/abs/1902.09130 (accessed January 12, 2021)

[22] Esfandiari H Newell R Anglin C Street J Hodgson AJ. A deep learning framework for segmentation and pose estimation of pedicle screw implants based on C-arm fluoroscopy. Int J Comput Assist Radiol Surg. (2018) 13:1269–82. 10.1007/s11548-018-1776-9 29808466

[23] Liebmann F Roner S von Atzigen M Scaramuzza D Sutter R Snedeker J. Pedicle screw navigation using surface digitization on the Microsoft HoloLens. Int J Comput Assist Radiol Surg. (2019) 14:1157–65. 10.1007/s11548-019-01973-7 30993519

[24] Maffulli N Rodriguez HC Stone IW Nam A Song A Gupta M. Artificial intelligence and machine learning in orthopedic surgery: a systematic review protocol. J Orthop Surg. (2020) 15:478. 10.1186/s13018-020-02002-z 33076945 PMC7570027

[25] Deib G Johnson A Unberath M Yu K Andress S Qian L. Image guided percutaneous spine procedures using an optical see-through head mounted display: proof of concept and rationale. J NeuroInterventional Surg. (2018) 10:1187–91. 10.1136/neurintsurg-2017-013649 29848559

[26] Lipson SJ. Spinal-fusion surgery – advances and concerns. N Engl J Med. (2004) 350:643–4. 10.1056/NEJMp038162 14960739

[27] Vos T Barber RM Bell B Bertozzi-Villa A Biryukov S Bolliger I. Global, regional, and national incidence, prevalence, and years lived with disability for 301 acute and chronic diseases and injuries in 188 countries, 1990–2013: a systematic analysis for the global burden of disease study 2013. Lancet. (2015) 386:743–800. 10.1016/S0140-6736(15)60692-4 26063472 PMC4561509

[28] Picavet HSJ Schouten JSAG. Musculoskeletal pain in the netherlands: prevalences, consequences and risk groups, the DMC3-study. Pain. (2003) 102:167–78. 10.1016/s0304-3959(02)00372-x 12620608

[29] Murray CJL Vos T Lozano R Naghavi M Flaxman AD Michaud C. Disability-Adjusted Life Years (DALYs) for 291 diseases and injuries in 21 regions, 1990–2010: a systematic analysis for the global burden of disease study 2010. Lancet. (2012) 380:2197–223. 10.1016/S0140-6736(12)61690-0 23245608

[30] Raciborski F Gasik R Kłak A. Disorders of the spine. A Major Health and Social Problem. Reumatologia. (2016) 4:196–200. 10.5114/reum.2016.62474 27826174 PMC5090028

[31] Bailly F Foltz V Rozenberg S Fautrel B Gossec L. The impact of chronic low back pain is partly related to loss of social role: a qualitative study. Joint Bone Spine. (2015) 82:437–41. 10.1016/j.jbspin.2015.02.019 26431929

[32] Schofield DJ Shrestha RN Percival R Callander EJ Kelly SJ Passey ME. Early retirement and the fnancial assets of individuals with back problems. Eur Spine J. (2011) 20:731–6. 10.1007/s00586-010-1647-8 21132556 PMC3082667

[33] Koes BW van Tulder MW Thomas S. Diagnosis and treatment of low back pain. BMJ. (2006) 332:1430–4. 10.1136/bmj.332.7555.1430 16777886 PMC1479671

[34] van Tulder MW Koes BW Bouter LM. Conservative treatment of acute and chronic nonspecific low back pain: a systematic review of randomized controlled trials of the most common interventions. Spine. (1997) 22:2128–56. 10.1097/00007632-199709150-00012 9322325

[35] Deyo RA Nachemson A Mirza SK. Spinal-fusion surgery—the case for restraint. Spine J. (2004) 4:S138–42. 10.1016/j.spinee.2004.08.001 14960750

[36] Heini PF. The current treatment—a survey of osteoporotic fracture treatment. osteoporotic spine fractures: the spine surgeon's perspective. Osteoporos Int. (2005) 16:S85–92. 10.1007/s00198-004-1723-1 15365699

[37] Hee HT Majd ME Holt RT Pienkowski D. Better treatment of vertebral osteomyelitis using posterior stabilization and titanium mesh cages. J Spinal Disord Tech. (2002) 15:149–56; discussion 156. 10.1097/00024720-200204000-00010 11927825

[38] Maruyama T Takeshita K. Surgical treatment of scoliosis: a review of techniques currently applied. Scoliosis. (2008) 3:6. 10.1186/1748-7161-3-6 18423027 PMC2346456

[39] Mackel CE Jada A Samdani AF Stephen JH Bennett JT Baaj AA. A comprehensive review of the diagnosis and management of congenital scoliosis. Childs Nerv Syst. (2018) 34:2155–71. 10.1007/s00381-018-3915-6 30078055

[40] Esfandiari H. An Intraoperative Position Assessment System for Pedicle Screw Insertion Surgeries (PhD Thesis). The University of British Columbia, Vancouver, Canada (2020).

[41] Katonis P Christoforakis J Aligizakis AC Papadopoulos C Sapkas G Hadjipavlou A. Complications and problems related to pedicle screw fixation of the Spine. Clin Orthop. (2003) 411:86–94. 10.1097/01.blo.0000068761.86536.1d 12782863

[42] Gautschi OP Schatlo B Schaller K Tessitore E. Clinically relevant complications related to pedicle screw placement in thoracolumbar surgery and their management: a literature review of 35,630 pedicle screws. Neurosurg Focus. (2011) 31:E8. 10.3171/2011.7.FOCUS11168 21961871

[43] Nevzati E Marbacher S Soleman J Perrig WN Diepers M Khamis A. Accuracy of pedicle screw placement in the thoracic and lumbosacral spine using a conventional intraoperative fluoroscopy-guided technique: a national neurosurgical education and training center analysis of 1236 consecutive screws. World Neurosurg. (2014) 82:866–71.e2. 10.1016/j.wneu.2014.06.023 24954252

[44] Mummaneni PV Dhall SS Ondra SL Mummaneni VP Berven S. Pedicle subtraction osteotomy. Neurosurgery. (2008) 63(suppl_3):A171–6. 10.1227/01.NEU.0000325680.32776.82 18812921

[45] Hartl R Lam KS Wang J Korge A Kandziora F Audigé L. Worldwide survey on the use of navigation in spine surgery. World Neurosurg. (2013) 79:162–72. 10.1016/j.wneu.2012.03.011 22469525

[46] Kosmopoulos V Schizas C. Pedicle screw placement accuracy: a meta-analysis. Spine. (2007) 32:E111–20. 10.1097/01.brs.0000254048.79024.8b 17268254

[47] Gibaud B Forestier G Feldmann C Ferrigno G Gonçalves P Haidegger T. Toward a standard ontology of surgical process models. Int J Comput Assist Radiol Surg. (2018) 13:1397–408. 10.1007/s11548-018-1824-5 30006820

[48] Jannin P Morandi X. Surgical models for computer-assisted neurosurgery. Neuroimage. (2007) 37:783–91. 10.1016/j.neuroimage.2007.05.034 17613249

[49] Katić D Julliard C Wekerle AL Kenngott H Müller-Stich BP Dillmann R. LapOntoSPM: an ontology for laparoscopic surgeries and its application to surgical phase recognition. Int J Comput Assist Radiol Surg. (2015) 10:1427–34. 10.1007/s11548-015-1222-1 26062794

[50] Perrone R Nessi F Momi E Boriero F Capiluppi M Fiorini P. Ontology-based modular architecture for surgical autonomous robots. In: The Hamlyn Symposium on Medical Robotics. London, UK: Imperial College (2014).

[51] Nagy TD Haidegger T. A DVRK-based framework for surgical subtask automation. Acta Polytech Hung. (2019) 16:61. 10.12700/APH.16.8.2019.8.5

[52] MacKenzie L Ibbotson JA Cao CGL Lomax AJ. Hierarchical decomposition of laparoscopic surgery: a human factors approach to investigating the operating room environment. Minim Invasive Ther Allied Technol. (2001) 10:121–7. 10.1080/136457001753192222 16754003

[53] Neumuth T. Surgical Process Modeling Theory, Methods, and Applications (Habilitation Thesis). University of Leipzig, Leipzig, Germany (2012).

[54] Stein G Eysel P Scheyerer MJ. Expertise orthopädie und unfallchirurgie wirbelsäule. Georg Thieme Verlag. (2019) 1:2216. 10.1055/b-006-14953326606158

[55] Wang J Chen Y Hao S Peng X Hu L. Deep learning for sensor-based activity recognition: a survey. Pattern Recognit Lett. (2019) 119:3–11. 10.1016/j.patrec.2018.02.010

[56] Uemura M Jannin P Yamashita M Tomikawa M Akahoshi T Obata S. Procedural surgical skill assessment in laparoscopic training environments. Int J Comput Assist Radiol Surg. (2016) 11:543–52. 10.1007/s11548-015-1274-2 26253582

[57] Forestier G Petitjean F Riffaud L Jannin P. Automatic matching of surgeries to predict surgeons' next actions. Artif Intell Med. (2017) 81:3–11. 10.1016/j.artmed.2017.03.007 28343742

[58] Franke S Meixensberger J Neumuth T. Intervention time prediction from surgical low-level tasks. J Biomed Inform. (2013) 46:152–9. 10.1016/j.jbi.2012.10.002 23111119

[59] Neumuth T Mudunuri R Jannin P Meixensberger J Burgert O. SWAN-Suite: the tool landscape for surgical workflow analysis. In Paris; Berlin; Boston, MA: De Gruyter (2007). p. 199–204.

[60] Lalys F Riffaud L Morandi X Jannin P. Automatic phases recognition in pituitary surgeries by microscope images classification. In: Navab N Jannin P, editors. Information Processing in Computer-Assisted Interventions. Berlin, Heidelberg: Springer (2010). p. 34–44. (Lecture Notes in Computer Science).

[61] Bhatia B Oates T Xiao Y Hu P. Real-time identification of operating room state from video. In: Proceedings of the 19th national conference on Innovative applications of artificial intelligence - Volume 2. Vancouver, British Columbia, Canada: AAAI Press (2007). p. 1761–6. (IAAI'07).

[62] Nagy DÁ Rudas IJ Haidegger T. OntoFlow, a software tool for surgical workflow recording. In: 2018 IEEE 16th World Symposium on Applied Machine Intelligence and Informatics (SAMI) Herlany, Slovakia (2018). p. 000119–24.

[63] Katić D Wekerle AL Görtler J Spengler P Bodenstedt S Röhl S. Context-aware augmented reality in laparoscopic surgery. Comput Med Imaging Graph. (2013) 37:174–82. 10.1016/j.compmedimag.2013.03.003 23541864

